# A Scoping Review of Mathematical Models Used to Investigate the Role of Dogs in Chagas Disease Transmission

**DOI:** 10.3390/ani13040555

**Published:** 2023-02-05

**Authors:** Edem Fiatsonu, Rachel E. Busselman, Martial L. Ndeffo-Mbah

**Affiliations:** Department of Veterinary Integrative Biosciences, School of Veterinary Medicine and Biomedical Sciences, Texas A&M University, College Station, TX 77843, USA

**Keywords:** Canine Chagas disease, dynamic transmission model, *Trypanosoma cruzi*

## Abstract

**Simple Summary:**

Chagas disease- caused by the parasite *Trypanosoma cruzi* and vectored by triatomine insects (‘kissing bugs’)- poses a serious threat to human and dog health. Mathematical modeling is an important tool to assess control interventions and determine how different factors affect disease transmission. In the Chagas disease transmission cycle, the application of mathematical modeling techniques to study the role of dogs in disease epidemiology and control has not been fully understood. The purpose of this study was to review mathematical models that investigated the role of dogs in Chagas disease transmission. We examine the modeling approaches used, assess their contribution to understanding dogs’ role in Chagas disease transmission and control, and discuss their strengths and limitations. We identified ten peer-reviewed articles. Five of the ten reviewed articles focused on evaluating the effectiveness of intervention measures to control disease transmission and three focused on estimating disease transmission risk. The reviewed studies show that dogs are not only at high risk of infection but also amplify the spread of infections in endemic areas. Moreover, these studies have demonstrated that eliminating infected dogs from households or frequent use of insecticide could be sufficient to nearly interrupt disease transmission.

**Abstract:**

Chagas disease is a zoonotic vector-borne disease caused by the parasite *Trypanosoma cruzi*, which affects a variety of mammalian species across the Americas, including humans and dogs. Mathematical modeling has been widely used to investigate the transmission dynamics and control of vector-borne diseases. We performed a scoping review of mathematical models that investigated the role of dogs in *T. cruzi* transmission. We identified ten peer-reviewed papers that have explicitly modeled the role of dogs in Chagas transmission dynamics. We discuss the different methods employed in these studies, the different transmission metrics, disease transmission routes, and disease control strategies that have been considered and evaluated. In general, mathematical modeling studies have shown that dogs are not only at high risk of *T. cruzi* infection but are also major contributors to *T. cruzi* transmission to humans. Moreover, eliminating infected dogs from households or frequent use of insecticide was shown to be effective for curtailing *T. cruzi* transmission in both humans and dogs. However, when insecticide spraying is discontinued, *T. cruzi* infections in dogs were shown to return to their pre-spraying levels. We discuss the challenges and opportunities for future modeling studies to improve our understanding of Chagas disease transmission dynamics and control.

## 1. Introduction

Chagas disease (American Trypanosomiasis) is a zoonotic disease affecting millions of humans and animals in the Americas [1]. It is a vector-borne disease caused by the parasite *Trypanosoma cruzi* (Chagas, 1909), which causes severe cardiac and gastrointestinal disease in infected mammals, including humans and animals [2,3]. Chagas disease is vectored by triatomine insects (aka ‘kissing bugs’) and is primarily transmitted to hosts by infected triatomine fecal material when introduced to a bite wound during or after feeding. It can also be transmitted when the infected triatomine or fecal material is consumed by the hosts [2]. Other transmission routes include vertical transmission, either transplacental or through maternal milk, unprotected contact with infected body secretions or blood, blood transfusion, organ transplantation and laboratory accidents [4]. An estimated 8 million people are currently infected globally, with most locally acquired cases occurring in Latin America; over 10,000 people die every year from Chagas disease, and more than 25 million people are at risk of acquiring the disease [5,6]. According to the World Health Organization, vector control still remains the most useful intervention to prevent *T. cruzi* infections in endemic areas [5].

Domestic animals, like dogs (*Canis lupus familiaris* Linnaeus, 1758), have been shown to play a crucial role in the transmission of *T. cruzi* [7]. Dogs play an increasingly significant role in contemporary human societies throughout the world. While many dog owners keep them as pets and companion animals, others keep them to guard their properties against intruders, hunt, herd, and assist law enforcement [8]. Dogs may contribute to *T. cruzi* transmission in a variety of ways, including consuming infected triatomines, maintaining the transmission cycle of *T. cruzi* as primary/major host reservoirs, as well as contributing to increased triatomine bug populations as an important blood meal source [9]. In this regard, dogs may provide a link between the sylvatic, peridomestic, and domestic epidemiological transmission cycles, increasing the risk of human *T. cruzi* infections [1,10,11]. Moreover, the prevalence of *T. cruzi* infection in dogs has been observed to be much higher than in local human populations [10], and analyses of triatomine blood meals have shown that dogs can account for the majority of identified triatomine blood meals in different settings [12,13,14,15,16].

Mathematical modeling has increasingly become an important tool in the study of infectious disease epidemiology. Data-driven mathematical models can be used to better understand the impact and contribution of different drivers of infectious disease dynamics, test scientific hypotheses of disease transmission mechanisms, and answer disease control policy questions [17]. Well-parameterized mathematical models can be used to estimate key biological parameters and to test a variety of possible scenarios in computer simulation before applying them in the field [18,19]. Mathematical models can also be used to help formulate hypotheses, inform data collection and determine appropriate sample size for field trials.

One of the seminal mathematical models for infectious diseases was developed by Ross in 1908 and 1911 to describe the transmission dynamics of Malaria [20,21]. It later gave birth to the Ross-MacDonald theory for vector-borne disease, which has become a cornerstone in vector-borne disease epidemiology [22,23]. This mathematical modeling theory has vastly contributed to the development of quantitative theory of vector-borne disease transmission and to the quantitative foundations of epidemiology [22,24]. This includes the development of metrics for disease transmission, such as the basic reproductive number, R_0, which describes the expected number of hosts that would be infected by a single infected host in a completely susceptible population, and the Vectorial Capacity, which describes the potential intensity of transmission by vectors [22]. Since then, mathematical models have been used to investigate and improve our understanding of the dynamics and control of several vector-borne diseases, including Chagas disease. These mathematical models are generally called dynamic transmission models or mechanistic mathematical models. They are models capable of reproducing the direct and indirect effects that may arise from an infectious disease spread and control program. Contrary to static models, which assume a constant risk of infection, these models consider risk of infection as a function of the number of infectious individuals in the population at a given point in time. For example, if an intervention reduces the pool of infectiousness at a given point in time, then the risk to uninfected healthy individuals will decrease. That is, individuals not reached by the program can still benefit by experiencing a lower infection risk. This phenomenon which is paramount to infectious disease dynamics can only be captured by dynamic transmission models.

The objectives of this paper are: (1) to identify mathematical modeling studies that account for the role of dogs in Chagas disease transmission, (2) to examine the modeling approaches used to develop these Chagas dynamic transmission models, (3) to assess the contribution of these models to understand Chagas disease transmission and control, and (4) to identify and discuss limitations of existing models.

## 2. Methods

A scoping literature review was conducted to identify mathematical models that accounted for the dynamics of *T. cruzi* transmission among triatomine populations and hosts-specifically dogs-using academic databases, without restrictions on date of publication. Mathematical models that did not explicitly account for the role of dogs in the transmission of *T. cruzi* were excluded from our search.

### Literature Search

The PubMed and Google Scholar databases were searched using predefined keywords. The following combination of search terms were applied across both search engines: “Models, Theoretical”[Mesh] OR “mathematical model*”[tw] OR “dynamic model*”[tw] OR “mathematical modeling”[tw] OR “mathematic model”[tw] OR “mathematical”[tw] OR “modelling” OR “ predictive model*”[tw] OR “biomathematic*”[tw] OR “biomathematics”[tw] OR “biomath”[tw] OR “mathematic*”[tw] OR “SI model*”[tw] AND “Chagas Disease”[Mesh] OR “*Trypanosoma cruzi*”[tw] OR “American trypanosomiasis”[tw] OR “*T. cruzi* infection*”[tw] AND “Dogs”[Mesh]. Additionally, reference lists of included studies were reviewed but did not yield any additional articles. Our review focused on the type of mechanistic mathematical models used in modeling the role of dogs in the transmission of Chagas disease. We highlighted evidence provided by mathematical models on Chagas disease dynamics and control efforts.

Journal articles were eligible for inclusion if they met the following criteria: (1) written in English; (2) peer-reviewed; (3) published on or before 2022; (4) described a mechanistic mathematical model that accounted for Chagas disease transmission dynamics, and (5) explicitly accounted for the role of dogs in disease dynamics.

## 3. Results

### 3.1. Purpose of the Modeling Articles

**Figure 1:**
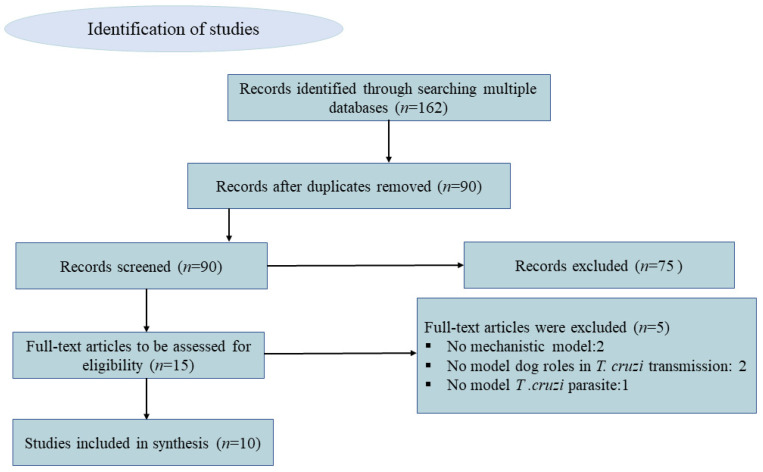
Conceptual diagram of the identification and selection process of articles for the inclusion in the scoping review using PRISMA (Preferred Reporting Items for Systematic review and Meta-Analysis).

In our scoping review of mathematical models of Chagas disease, we searched PubMed and Google Scholar databases and found 61 and 101 entries, respectively. After removing duplicates and reviewing titles and abstracts, 15 references appeared to meet our criteria. After full-text review, 5 were further excluded due to the following reasons: not proposing a mechanistic model (*n* = 2), not explicitly modeling dog roles (e.g., general hosts) (*n* = 2), and modeling other American Trypanosoma (*T. rangeli* Tejera, 1920) as an hypothetical causative agent of Chagas disease (*n* = 1) (see Figure 1).

Applying our exclusion criteria, we identified only 10 peer-reviewed publications that used mathematical models to explicitly model the roles of dogs in *T. cruzi* dynamics. These articles were published between 2001 and 2021. Six of these 10 articles assessed the impact and effectiveness of intervention measures for the control of *T. cruzi* transmission. These control interventions included the use of zooprophylaxis in households [25,26,27], insecticide spraying [28,29], and the World Health Organization (WHO) strategies for interrupting the transmission of *T. cruzi* in Latin America [30]. Two of the remaining four articles focused on describing the dynamics of *T. cruzi* infection at a household and population-level. The first paper used modeling to estimate the initial spread of infection in a typical rural household in the endemic zone [31]. The second focused on characterizing the reproduction number (R_0_) and several types of reproductive numbers (T) for relatively important hosts and estimating the expected number of new infections generated by different routes of *T. cruzi* transmission [32]. Of the remaining two articles, one developed a model to investigate the significance of the method of Chagas disease transmission, including oral transmission due to predation and congenital transmission relative to vector biting [33], and the other compared two compartmental models evaluating disease transmission dynamics by comparing the resulting seroprevalence estimates of the models to the historical seroprevalence data [34]. Out of the ten identified articles, one was published in 2001 [26], and the remaining nine were published after 2010. This indicates mathematical models are increasingly being used to study Chagas disease dynamics and the role of dogs in *T. cruzi* transmission.

### 3.2. Mathematical Models

Out of the 10 modeling articles included in this study, 9 used equation-based (aka deterministic) model paradigms to model the dynamics of Chagas disease among triatomines and hosts. The use of a set of differential equations that divides the population into compartments is the most common type of equation-based model [35,36]. In the compartmental model, the population is assumed to be homogeneous, well-mixed and can be subdivided into compartments depending on the disease status and transmission dynamics [37,38]. In this review, the most frequently used models are the one that were divided into susceptible, *S*, and infected, *I*, compartments (9/10). In these compartmental models, susceptible hosts become infected through contact with infected vectors, and susceptible vectors become infected by contacting infected hosts. Once infected the host or the vector will remain infected for the rest of their life. This type of model is called SI (Susceptible-Infected) model. In SI-type models, the infected stage is either aggregated into a single compartment, *I*, or it can be distributed into several sub-compartments representing different stages of disease progression, such as acute, chronic indeterminate, and chronic with determinate pathology. Out of the 9 Chagas disease compartmental models, 4 did not explicitly account for the various human infection stages [27,28,29,33], while the remaining 5 did [25,30,31,32,34].

Only one out of 10 research articles used an agent-based model (ABM) [26]. ABMs are computer simulations with a set of coded rules that comprise agents (e.g., households, individuals, government, any entity of interest) that interact with each other and the environment. ABMs capture the aggregation of individual behaviors and the ability of the agents to make their own decisions in the model per the set of rules given to them [37]. They are also used to capture the transmission dynamics of infectious disease together with the heterogeneous, stochastic nature of all agents’ populations. In Chagas disease modeling, the ABM was used to make it more convenient to capture the age structure in human households, the small numbers of domestic vertebrates (dogs and chickens), and the nymphs and adult stages of the triatomines to understand *T. cruzi* transmission.

### 3.3. Transmission Routes of Trypanosoma cruzi

The transmission of the parasite *T. cruzi* is categorized into two main routes: vectorial and non-vectorial. In the vectorial transmission route, *T. cruzi* can be spread through stercorarian (vector-fecal) transmission, which is when a triatomine excretes the parasite in its fecal material, which then enters a host (mammals, including humans and animals) through the bite wound, mucous membrane, or a break in the skin. Another vector transmission route is oral transmission, which occurs after consumption of fruit or juice that has been contaminated with infected triatomine feces by a host (humans and animals) or consumption of infected triatomines by animals (including dogs). Non-vectorial transmission routes of *T. cruzi* include transplacental and transmammary transmission from infected mother to offspring in both humans and animals, blood transfusions, and uncommon laboratory accidents in humans. The *T. cruzi* transmission routes included in a Chagas disease transmission model depend on the questions the researchers are trying to answer and more importantly on the availability of data.

All 10 articles accounted for vectorial transmission in humans and dogs. Five of the 10 articles accounted for congenital transfusion transmission in humans and/or dogs [25,30,31,32,33]. Only one article accounted for three transmission routes in dogs: vectorial, congenital, and oral transmission due to predation [33]. A summary of the modeled transmission routes is provided in Table 1.

### 3.4. Model Parameterization

One of the greatest challenges with modeling infectious diseases or outbreaks is what is known as “parameterizing” the model. Parameterizing models is the process of selecting or estimating the values of all the parameters that describe the dynamics of the disease being investigated. Some of these key parameters are transmissibility rates, birth and death rates, infection duration, contact rates between hosts and vectors, and other parameters that are of interest and play key roles in infectious disease dynamics. These parameter values must be specified or estimated before modeling disease dynamics scenarios. Well-parameterized mathematical models allow modelers to experiment with a variety of possible scenarios of control interventions in computer simulations before applying to reality.

Only four out of the 10 articles calibrated their model to empirical data [26,30,31,34]. Lee et al. [30] populated and calibrated their model to describe *T. cruzi* transmission in a rural village in Yucatan, Mexico. Their model was calibrated under the assumption of a 32.5% prevalence in triatomines and a seroprevalence estimate of 1.85% in humans and 14.58% in dogs. The various transmission probabilities were estimated through model calibration, including from triatomines to humans and dogs, from dogs and humans to triatomines, transmission across the infection stages in humans (acute, indeterminate/chronic stage) to triatomines, congenital transmission in hosts, as well as the triatomine feeding proportions across hosts in domestic and peridomestic settings. Other important parameters calibrated in their models included the transmission risk through infected blood transfusion or organ transplant, and relative prevalence of infection among women of reproductive age as compared to the general population. The authors provided point value estimates as well as calibrated value intervals representing the parameters’ minimum and maximum values that meet the targeted baseline outputs. Bartsch et al. [34] calibrated two models (the PHICOR/CIMDA and the Princeton model) using 10-year age-structured *T. cruzi* seroprevalence data of entomological surveillance for triatomine bugs and infected houses from the national Chagas Disease Control Programme collected by the Venezuelan Ministry of Health between 1958 and 1998. The PHICOR/CIMDA used only three age groups, while the Princeton model used six age groups. The observables of interest were age-stratified *T. cruzi* seroprevalence in humans and triatomines. Fabrizio et al. [31] calibrated their model to Buenos Aires City (Argentina) with the assumption that 3.7% of the human population was infected with *T. cruzi*, with a 4.4% prevalence in pregnant women and 2.94% prevalence in blood donors. Cohen and Gurtler [26] calibrated their model based on comprehensive household data from 65 houses in three villages (Amama, Trinidad and Mercedes) in Northern Argentina. The median household consisted of 5 people, 3 infected dogs, 1 cat and 8–27 chickens and ducks. The model was used to approximate the ratio of feeding dogs and chickens to humans as 3. This was done as a rough midpoint value for a wide range of variations in empirical estimates. Their model was used to predict infections among humans, chickens and dogs in a household as well as the number of infected and uninfected bugs, and the seasonal distribution of feeding contacts [26]. The remaining six articles did not calibrate their models to empirical epidemiological data, but simply parameterized them with parameter values from the literature [25,27,28,29,32,33].

### 3.5. Reproductive Numbers

A very important threshold quantity is the basic reproduction number, sometimes called the basic reproductive number or basic reproductive ratio [39], which is usually denoted by R_0_. The basic reproductive number is the average number of secondary infections produced when one infected individual is introduced into a totally susceptible population. This concept is fundamental to the study of infectious diseases, epidemiology, and within-host pathogen dynamics. The value of R_0_ determines whether the disease will vanish in the population or will persist. When R_0_ is greater than 1, disease outbreaks will occur and the pathogen will be established in the population; otherwise, the disease disappears. Mathematical modeling provides valuable help in quantifying disease control possibilities by examining key disease aspects, determining threshold quantities, and evaluating the effectiveness of control strategies.

When the control intervention programs are targeted at a specific host type or subset of host types, R_0_ is not a good indicator of the required control efforts. The type-reproduction number, denoted *T*, was proposed as a metric for targeted control programs [40,41]. The type-reproduction number for a target subset of a population (or subset of host) represents the average number of secondary cases in this subset produced by primary cases in the same subset in a completely susceptible population, either directly or passing through other types of host. It is a threshold quantity that is used to correctly determine the critical control effort to interrupt disease transmission in a heterogeneous population when a control strategy is targeted at a particular subset of hosts [41]. Insecticide spraying is such a targeted control measure that focuses on triatomine vectors. From the properties of the type-reproduction number, it was shown that in a multi-host system, *T* ≥ 1 indicates the presence of a reservoir host other than the targeted host/subset of host [40]. In other words, *T* ≥ 1 indicates achieving 100% control of the targeted host (subset of host) should not be enough to interrupt disease transmission.

Three out of the 10 studies estimated R_0_ with values ranging between 1.10 and 8.04 across endemic settings [25,27,32]. Cruz-Pacheco et al. [25] estimated the basic reproductive number to be R_0_ = 5.8, the human reproductive number (the average number of secondary infections that result from an infected individual within only a human-vector cycle) as R_h_ = 1.3 and transmitters (dog) reproductive number (accumulated secondary infections generated from an infected individual within only a dog-vector cycle) as 11.4. This indicates that an infected dog may generate eight times more new infected cases than an infected human.

Fabrizio et al. [32] estimated the basic reproductive number to be R_0_ = 5.6. They showed that one infected human infects 21 triatomines, 100 infected triatomines are necessary to infect one human, 34 infected triatomines are necessary to infect a dog, and that each dog infects on average one triatomine per day [32]. These results show that dogs are a major contributor to Chagas disease transmission. Fabrizio et al. [32] also estimated the type- reproduction number, *T,* for humans, triatomines, and dogs and showed them to be equal to 5.7, 0.1, and 0.51, respectively. As the *T* value for humans is greater than 1 (5.7), it indicates the existence of *T. cruzi* reservoir (dogs or triatomines) other than human [32,41]. This reservoir contributes significantly to disease spread among humans, and interventions not targeted to that reservoir will not be able to control *T. cruzi* transmission. The results for triatomines and dogs indicate more than 97% of triatomines should remain uninfected or 92% of dogs should remain permanently protected from infection to eliminate *T. cruzi* transmission in a given location. However, interrupting human *T. cruzi* transmission alone will be insufficient to interrupt disease transmission because *T. cruzi* transmission is primarily sustained by non-human host reservoirs (e.g., dogs) [32].

### 3.6. Key Outcomes of the Modeling Studies

Fabrizio et al. [32] and Cruz-Pacheco et al. [25] have shown that R_0_ for Chagas disease is very sensitive to the death rates and transmission rate between infected triatomines and dogs. Specifically, Cruz-Pacheco et al. [25] showed that if triatomine mortality increases by a factor θ > 1, R_0_ decreases by a factor 1/θ. They concluded that if an effective insecticide is applied every three months, almost all bugs will ultimately be eliminated from the controlled area. In addition, they computed the basic reproduction number to be 5.8. Therefore, Chagas disease can be eliminated from an endemic area if 1/θ*5.8 < 1 or 5.8 < θ, meaning that vector mortality should be increased more than six times to reduce R_0_ below 1. This could be achieved if an insecticide is sprayed at least once every month [25]. In another study, Flores-Ferrer et al. [27] estimated the R_0_ value for dogs to be 1.10, indicating that dogs are one of the pathogen’s reservoirs, and the local transmission cycle of *T. cruzi* will persists in an endemic village even in the absence of vector immigration from the sylvatic habitat. Moreover, they showed that reducing the dog population and increasing non-competent hosts will lower new human infections by up to 56% and 39%, respectively [27]. Coffield et al. [33] evaluated the related contribution of oral consumption and vector biting to dog infections. They showed that although dogs have a higher likelihood of infection by the oral consumption of vectors than by vector bites, most dogs become infected via biting without oral transmission, and increasing the dog’s oral consumption rates of triatomine will only moderately increase the number of infected dogs [33].

Spagnuolo et al. [29] showed that when the blood meal supply is abundant, though insecticide spraying may cause dramatic reduction of *T. cruzi* infection among dogs and triatomines in the short term, disease prevalence will rapidly return to pre-control era levels following interruption of insecticide spraying. This dramatic change in disease outcome supports the need for continuous insecticide spraying in Chagas endemic areas to maintain disease control gain from previous control efforts. However, this study does account for the potential emergence of insecticide resistance and its impact on insecticide efficacy and disease transmission among others. Another modeling study, Spagnuolo et al. [28] showed that triatomine population and Chagas disease prevalence can return to their pre-spraying levels in approximately 5–8 years if pesticide use is discontinued. However, they showed the impact of pesticide use varies significantly with the abundance of blood supply [28]. For example, they showed that the number of triatomines and infected dogs more than tripled with a readily available blood supply compared to the baseline model with limited blood supply.

Limiting transmission of *T. cruzi* infections is the strategy to achieve the 2020 Sustainable Development Goals of 100% interruption and control of domestic, congenital, and transfusion transmission in Chagas endemic regions [30]. In evaluating this strategy, Lee et al. [30] showed that interrupting transmissions from vector to dog by 100% would have a relatively significant impact on the rate of new infection among humans. Similar results were obtained by Chen et al. [37], which showed that in rural settings, keeping dogs and other highly infectious vertebrates out of bedroom areas can effectively reduce the vector and human prevalence rates. However, eliminating infected dogs from a household with infected people is nearly sufficient to interrupt the transmission of *T. cruzi* [37]. Key results of the identified modeling studies are summarized in Table S1.

## 4. Discussion

The results of our review indicate that mathematical models are increasingly being used to model Chagas disease dynamics, which would enable good policy decisions in a variety of settings. To our knowledge, this is the first review that identifies and compares mathematical models used to model *T. cruzi* infection among triatomines and dogs. These models explicitly account for the role dogs play in the transmission of *T. cruzi*. The models were used to explore the transmission dynamics of *T. cruzi* infections and the impact of alternative public health policy options for disease control. The reviewed studies showed that dogs are not only at high risk of infection but also amplify the spread of infections in endemic areas. Moreover, they showed that eliminating infected dogs from households or frequent use of insecticide could be sufficient to nearly interrupt disease transmission.

This review, like any other scoping literature review, has the limitation of only being as reliable as the methods used to estimate the effect in each primary modeling paper. Although there was no restriction placed on publication date and geographical location, the publication language was restricted to English. Given that Spanish and Portuguese are the predominant languages in Latin America, which is the main geographical region for Chagas disease, our review is limited by not considering articles published in those languages. The framework for this review was built to provide an overall overview of the peer-reviewed literature that captures the roles of dogs in the transmission of Chagas disease using a mathematical modeling approach. Therefore, it did not account for literature that described other Chagas disease modeling approaches, such as statistical modeling methods for simulating disease transmission or evaluating transmission risk.

Several research gaps were identified in this review. For example, none of the publications focused on modeling canine Chagas disease, which is an important veterinary public health problem. Future modeling studies should investigate canine Chagas disease transmission dynamics. These will help improve our understanding of the dynamics of *T. cruzi* infections in different dog populations (e.g., working dogs, pets, and stray dogs) and identify optimal intervention strategies for disease control. Such models would be pertinent in locations where transmission occurs primarily in the peridomestic environment and canine Chagas disease is prevalent, such as in the southern United States, where Chagas disease is endemic and there is a shared border with a hyperendemic zone, like Mexico. The impact of migration of triatomines between the domestic and peridomestic settings could have a significant implication on the control intervention of triatomines; however, that was not explored in any of the modeling articles identified. Few models included the peridomestic environment or triatomine migration rates, and none considered the potential impacts of seasonality on the triatomine maturation rate or transmission rate on *T. cruzi* infections among triatomines and dogs.

In addition, the modeling articles that we reviewed had limited or no access to empirical data to inform the key model parameters. It is difficult to collect data on some key triatomine model parameters, like migration rate, movement between the domestic and sylvatic environment, and difficulties in diagnosing *T. cruzi* infections. As a neglected tropical disease, Chagas has received relatively little attention and public health resources for adequate disease surveillance and control efforts. As a result of these, there are only limited epidemiological and entomological data available for most endemic regions. Empirical data for key model parameters such as triatomine migration rate, infection incidence among dogs within specific endemic areas, and triatomine population density, are not available. Consequently, the majority of Chagas disease models were not fitted or calibrated to observed data, and some essential model parameter values were based on assumptions or were parameterized using values from different endemic regions [25,27,28,29,32,33]. This lack of data may limit modelers from including some key ecological or biological parameters in their model, which may influence the model’s predictive ability. Another key limitation of some of the reviewed articles is the fact that these models did not explicitly differentiate between *T. cruzi* transmission settings, such as peridomestic versus sylvatic transmission environments. Ignoring differences between these transmission settings could greatly over- or underestimate the role of dogs in the transmission dynamics of *T. cruzi* and the impact of vector control interventions.

Detailed high-quality epidemiological and entomological data on Chagas disease must become more readily available and accessible to modelers to develop more accurate and informative models of *T. cruzi* transmission in different endemic areas. Such a dataset will be paramount for the development of more accurate models for Chagas disease. In addition to better data quality, integrated multidisciplinary collaboration between public health practitioners, field entomologists and epidemiologists, and mathematical modelers is needed to develop more realistic epidemiological models of Chagas disease for both humans and animals.

## 5. Conclusions

Mathematical modeling has been used to understand the transmission dynamics of *T. cruzi* infections and the epidemiological impact of future vector control strategies, such as insecticide spraying or diverting triatomine to non-transmitter hosts like chickens, in different settings. It has also proved important for exploring potential combinations of strategies for interrupting the transmission of *T. cruzi* to achieve Chagas disease elimination. As dogs are increasingly recognized as key hosts of *T. cruzi* transmission, further modeling investigations that can account for many of their potential roles in transmission are warranted. Modeling should be integral to the design and implementation of future vector control strategies such as insecticides spraying, host-targeted (i.e., dogs) insecticide treatments, or novel vector control tools. More specifically, mathematical models could be developed to identify and evaluate optimal treatment regimens for canine oral systemic insecticides, which are used to prevent tick and flea infestations and have been shown to be effective at inducing mortality in triatomines. Though oral systemic insecticides are widely recommended for use in dogs, it remains unclear whether the optimal treatment regimen for canine Chagas disease control may vary with the disease transmission settings.

## Figures and Tables

**Table 1:** Summary of transmission routes and the populations involved.

Transmission Routes	Population Involved	Settings	Author
Vectorial	Triatomines, humans & dogs	Domestic & Peridomestic	Cohen & Gurtler [26], Flores-Ferre et al. [27], Spagnuolo et al. [28,29], Lee et al. [30]
		Domestic	Cruz-Pacheco et al. [25], Coffield Jr et al. [33], Fabrizio et al. [31,32], Bartsch et al. [34]
Congenital	Humans and dogs	Domestic & Peridomestic	Lee et al. [30]
	Humans only	Domestic	Cruz-Pacheco et al. [25], Coffield Jr et al. [33], Fabrizio et al. [31,32]
Transfusion	Humans only	Domestic & Peridomestic	Lee et al. [30]
		Domestic	Fabrizio et al. [31,32]
Oral consumption	Dogs only	Domestic	Coffield Jr et al. [33]

## Data Availability

Not applicable.

## Supplementary Materials

The following supporting information can be downloaded at: https://www.mdpi.com/article/10.3390/ani13040555/s1, File S1-Table S1: Summary of Chagas disease mathematical models that explicitly accounted for dogs’ contribution in *T. cruzi* transmission.
