# Supplementary material for: A Scoping Review of Mathematical Models Used to Investigate the Role of Dogs in Chagas Disease Transmission
Source: Animals (Basel). 2023 Feb 5;13(4):555. doi: 10.3390/ani13040555 (PMC9951694; doi:10.3390/ani13040555)
Supplement: Supplementary file 1 [file animals-13-00555-s001.zip › animals-2170984-supplementary.pdf]

Table S1. Summary of Chagas disease mathematical models that explicitly accounted for dogs' contribution in *T. cruzi* transmission.

Year	Author	Model used	Objectives	Results
2001	Cohen & Gurtler [26]	Agent-based model that captures the age structure of a household's human population, dogs and chickens, triatomines, and transmission seasonality during spring and summer was used.	The model is used to evaluate the effectiveness of control measures to interrupt disease transmission.	The model shows that eliminating infected dogs from a household with infected people is nearly sufficient to interrupt the transmission of *T. cruzi*.
2011	Spagnuolo et al. [28]	Deterministic SI-model that captures the dynamics of *T. cruzi* among humans, triatomines, dogs and chickens was used. The model includes the seasonal temperature and seasonal biting rate.	The model is used to evaluate the effectiveness of pesticide use to control Chagas disease.	It predicts that vector populations and the disease can return to their pre-spraying level within 5-8 years if pesticide use is discontinued. Moreover, the number of triatomines and infected dogs increase with available triatomine blood supply.
2012	Spagnuolo et al. [29]	A delayed logistics-typed SI-model that captures the dynamics of *T. cruzi* transmission among humans, triatomines, dogs and chickens was used. It accounts for a delayed seasonal biting rate and includes triatomine death rate as a periodic function (natural death and spraying induced-death)	The model is used to evaluate the impact of insecticide use for the control of Chagas disease.	The modelIt shows that annual spraying with insecticides causes a rapid decrease in infected dogs. If insecticide spraying is discontinued, dog infections will return to the pre-spraying values within approximately 7 years.
2012	Cruz-Pacheco et al. [25]	Deterministic SI-model that captures *T. cruzi* transmission among triatomines, humans, transmitters (dogs, cats) and non-transmitters (chickens, birds) was used.	The next-generation matrix is used to compute the basic reproduction number R_0, human reproductive number R_h and transmitter (e.g. dog) reproductive number R_t.	The values for R_0, R_h and R_t were estimated to be 5.8, 1.3 and 11.4, respectively. This result indicates that transmitters (dogs) are primary drivers of Chagas transmission. Moreover, the analysis showed that insecticide should be sprayed at least once a month to interrupt disease transmission.
2013	Coffield Jr et al. [33]	A deterministic SI-model that captures the dynamics of *T. cruzi* infection among humans, triatomines, dogs and chickens was used. It accounts for oral transmission due to predation and congenital transmission in infected humans and dogs.	The model was used to analyze the dynamics of Chagas disease in the presence of multiple transmission routes.	The study showed that even in the presence of low vectors' biting preference for dogs, dogs still become sufficiently infected via oral transmission to drive infection in triatomines and humans.

2014	Fabrizio et al. [31]	A deterministic SI-model that captures three human infection stages (acute, chronic indeterminate, and chronic), susceptible and infected triatomines, and dogs was used.	The model was used to estimate the number of new infections generated by an infected individual through each transmission route at each disease stage, and analyze the sensitivity the basic reproduction number to model parameters.	Model simulation shows dogs are at high risk of infection with *T. cruzi* even in settings where the pathogen is newly introduced. For example, it shows that it takes only 19 days for 80 initially infected triatomines to infect a dog in a totally susceptible dog population.
2016	Fabrizio et al. [32]	A deterministic SI-model that captures three human infection stages (acute, chronic indeterminate, and chronic), susceptible and infected triatomines, and dogs was used.	Next-generation matrix is used to analyze the SI model captures three human infection stages, susceptible and infected triatomines, and dogs	The model estimated R_0 value to be equal to 5.6, and showed that R_0 value is very sensitive to the death rates and rates of transmission between infected triatomines and dogs. Moreover, it showed that one hundred infected triatomines introduced into a totally susceptible population will generate an average of three infected dogs, and one infected dog will generate one infected triatomine per day. This result indicates that dogs are amplifiers of *T. cruzi* transmission.
2017	Bartsch et al. [34]	Two deterministic SI-type models are considered: 1)The PHICOR/CIDMA represents triatomines, humans (three general age categories) and dogs involved in *T. cruzi* transmission; 2)The Princeton model only represents vector (uninfected, exposed and incubating, and infected) and human (six ten-year age categories) populations involved in *T. cruzi* transmission.	The paper compares two models that were developed to simulate Chagas disease dynamics in Latin America	Both models were fitted to 10-year age-specific *T. cruzi* seroprevalence data from Venezuela and were shown to generate predictions that accurately mimic age-specific seroprevalence data for the next 10-year period according to the historical data.
2018	Lee et al. [30]	A deterministic SI-model that captures the dynamics of *T. cruzi* among humans, triatomines, dogs and chickens in both domestic and peridomestic habitats. The model accounted for three transmission routes: vector-borne, congenital, and transfusional *T. cruzi* transmission.	The model was used to evaluate the impact of interrupting each transmission route and fully implementing the WHO Chagas elimination strategy.	Vectorial transmission was shown to account for up to 83% of new infections, and interrupting all three transmission routes could reduce new human infections by up to 90% over a five-year period.

2019	Flores-Ferre et al. [27]	Deterministic SI-models that capture the dynamics of *T. cruzi* in a community of competent (human, dogs, cats and rodents) and non-competent (avian) host species.	The model was used to analyze the dynamics of *T. cruzi* transmission in a multi-host (synanthropic and domesticated species) community.	The model estimated R_0 to be equal to 1.10 ($R_0 > 1$) for dogs, indicating that dogs are a reservoir of *T. cruzi* and the transmission cycle of *T. cruzi* can persist in Chagas endemic village even in the absence of vector immigration from the sylvatic habitat.
